# Comparison of Dentinal Tubule Penetration between a Calcium Silicate-Based Sealer with Ultrasonic Activation and an Epoxy Resin-Based Sealer: A Study Using Confocal Laser Scanning Microscopy

**DOI:** 10.1055/s-0041-1735429

**Published:** 2021-10-21

**Authors:** Dani Song, Sung-Eun Yang

**Affiliations:** 1 Department of Conservative Dentistry, Seoul St. Mary’s Hospital, College of Medicine, The Catholic University of Korea, Seoul, Republic of Korea

**Keywords:** calcium silicate-based sealer, epoxy resin-based sealer, CLSM analysis

## Abstract

**Objective** The aim of this study was to compare the degree of dentinal penetration between an epoxy resin-based sealer applied by using two different filling methods and an ultrasonically activated calcium silicate-based sealer via confocal laser scanning microscopy (CLSM).

**Materials and Methods** Forty-five extracted permanent maxillary premolars with type II canals (Vertucci’s classification) were subjected to the experiment. The root canals were instrumented and distributed randomly into the following three groups: AH Plus + continuous wave technique (AHC group); AH Plus + single cone technique (AHS group); and Endoseal MTA + single cone technique with ultrasonic activation (EMS) group. Each sealer was labeled with rhodamine B dye to allow visualization under CLSM. The sealer penetration depth in each sample was observed at 2 mm and 5 mm from the apex by using CLSM. The data were statistically analyzed by using analysis of variance or Kruskal–Wallis H test according to normality of variable (α = 0.05).

**Results** In all groups, the maximum sealer penetration depth, mean fluorescence intensity, and sum fluorescence intensity values were higher at the 5-mm level than at the 2-mm level. At the 5-mm level, the EMS group showed the lowest value ( *p* = 0.02). At the 2-mm level, there were no statistically significant differences among any of the groups. The AHC group showed higher values than the other groups, but there was no statistically significant difference in the apical area where access of instruments was difficult.

**Conclusion** The AHC group showed the highest dentinal tubule penetration, but had questionable filing efficacy in the apical area, which is of particular importance for the success of root canal treatment. Therefore, in areas such as the apical 2 mm of premolars with type II canals, which are difficult to access by using instruments such as heat carriers, other appropriate approaches may be required accordingly.

## Introduction

The aim of root canal filling is to prevent reinfection of the shaped and disinfected root canal. Complete canal obturation is important for this purpose, and the sealer plays an important role in minimizing the space between the gutta percha (GP) and the canal wall. Root canal sealers are used to fill complex spaces such as the space between the GP cone and the root canal wall, isthmuses, ramifications, deltas, and accessory canals. Since root canal sealers play an important role in canal filling, efforts have been made in recent years to develop various sealers along with obturation methods.

Obturation of the root canal has been done by using various techniques. The continuous wave technique—which is a sort of warm vertical GP technique—is a method of applying pressure after softening the GP by applying a heat source to the master cone and shows good sealing ability. 1 The single cone technique is a method of filling root canal with one gutta-percha cone of the same size and taper as the root canal preparation and a sealer. 2 It is an easy and inexpensive method because there is no need for a separate heat carrier or back filling device.

Epoxy resin-based sealers such as AH Plus (Dentsply DeTrey, Konstanz, Germany) are known to have excellent sealing properties and favorable mechanical properties. 3 However, they have the disadvantages of low flowability and high cytotoxicity, especially before setting and the flow of endodontic sealers may determine how effectively they can obturate complex spaces observed in the root canals. 4 5 6 7 8

Newly developed calcium silicate-based sealers have good flowability, low cytotoxicity, and high biocompatibility. 9 10 They have bioactivity on the surface of the material and induce bone formation. Some recent studies have shown that these sealers were comparable to resin-based sealers in terms of sealing ability, but long-term studies are inadequate. 11

Tight sealing efficacy can be obtained through high adaptability of the filling materials. 12 The better the penetration of sealer into the dentinal tubule, the higher the adaptability between filling material and canal wall can be considered. Calcium silicate-based sealers are reported to penetrate tubules to a comparable extent to resin-based sealers. 11

Sealer penetration into the dentinal tubules can be evaluated by using several methods. Of these methods, confocal laser scanning microscopy (CLSM) is especially useful because it can clearly visualize infiltration of the sealer tag into dentinal tubule with few artifacts. 13 14 15

The purpose of this study was to compare the dentinal tubule penetration of an epoxy resin-based sealer with two different filling methods and an ultrasonically activated calcium silicate-based sealer by using CLSM. The null hypothesis of this study was as follows: there might be no difference in penetration depth into dentinal tubule, especially at 2-mm level among groups tested.

## Materials and Methods

### Sample Preparation

This study was approved by the institutional review board of Seoul St. Mary’s Hospital, the Catholic University of Korea (KC18TNSI0694). Forty-five freshly extracted, permanent maxillary premolars of type II canals (Vertucci classification) 16 were selected and stored in sterile saline solution. A radiographic examination was conducted of the buccal and proximal aspects of each tooth to check the canal morphology, and teeth with severe root curvature (> 25 degrees, measured by the Schneider’s method), 17 canal obliteration, and a total crown-root length under 15 mm or over 23 mm were excluded. The teeth samples were stored in distilled water until the access opening was made.

The access opening was prepared with a #4 round bur and an Endo-Z bur (Dentsply Maillefer, Ballaigues, Switzerland) on each tooth. A size 10 K-file (Dentsply Maillefer) was placed in the root canal until it reached the apex, and the working length was then determined by subtracting 1 mm from this length. To unify the apical size, all roots were cleaned and shaped by using nickel-titanium rotary files (ProTaper F1 [Dentsply Maillefer] and Profile size 30/0.06 [Dentsply Maillefer]) in the presence of a 2.5% NaOCl solution.

After completing preparation, the canal was irrigated with 5 mL of 17% EDTA and 5 mL of 2.5% NaOCl solution with a 28-gauge side-vented irrigation needle (Monoject endodontic needle; Tokyo/Kendall, Mansfield, Massachusetts, United States). For passive ultrasonic irrigation, an Irrisafe tip (NSK, Tokyo, Japan) set at power level 2 was applied in an in-and-out motion up to two-thirds of the length of the canal for a total of 1 minute. The root canals were then dried with paper points (DiaDent, Cheongju, Korea).

### Classification of the Groups

#### The Samples were Randomly Divided into Three Groups (n = 15 Each)

AH Plus + continuous wave technique (AHC group): AH Plus sealer was prepared according to the manufacturer’s instructions. The sealer was labeled with a few grains of 0.1% rhodamine B (Sigma–Aldrich, St. Louis, Missouri, United States). After applying a sealer coating at the end of the master GP cone (size 30/0.06) (DiaDent), the GP cone was slowly inserted into the canals. The inserted GP cone was cut and down-packed to 4 mm from the WL with System B (Kerr Endodontics, Orange, California, United States) set to 200°C according to the manufacturer’s instructions. After maintaining apical pressure on the apical plug of GP for 10 seconds, the canal was backfilled by using Obtura (Obtura Spartan Endodontics, Algonquin, Illinois, United States).

AH Plus + single cone technique (AHS group): After thoroughly applying the sealer coated at the end of the master cone in an up-and-down motion, the cone was inserted into the root canal. The excess core was trimmed off at the canal orifice level by using System B.

Endoseal MTA + single cone technique with ultrasonic activation (EMS group): A coated GP cone was put into the root canal. According to the manufacturer’s instructions, the ultrasonic activation was delivered directly to the dental pincette for 3 seconds by using an ultrasonic tip (StartX #3, Dentsply Maillefer) connected to the ultrasonic device (P-5 Newtron XS; Satelec, Mount Laurel, New Jersey, United States). Power was set on “3” in the green mode. The excess GP was cut off at the canal orifice level by using System B.

#### Confocal Laser Scanning Microscopy Evaluation

Each sample was filled with cotton pellets and temporary material (Caviton; GC., Tokyo, Japan). For complete setting of the sealer, samples were stored at 37°C and 100% humidity for 7 days. The specimens were placed in a plastic mold (SHINJECT; Shinhung, Seoul, Korea) and embedded with acrylic resin (Tokuso; Tokuyama, Tokyo, Japan). The specimens were then sectioned by using a low-speed diamond saw (NTI-Kahla, Kahla, Germany) perpendicular to the longitudinal axis at 2 or 5 mm from the apex to represent the apical and middle thirds, and were approximately 1 mm thick.

The prepared specimens were mounted on a slice glass and observed with a CLSM (LSM 800; Carl Zeiss Microscopy, Jena, Germany) with ×5 magnification. Each image was evaluated by using ZEN software (Carl Zeiss Microscopy). First, the maximum sealer penetration depth into the dentinal tubules was measured by using ZEN software. The penetration percentage of the sealer was then evaluated. 18 The mean fluorescence intensity (MFI) and sum fluorescence intensity (SFI) were analyzed by ZEN software. The MFI and SFI refer to the mean and sum of the areas infiltrated by rhodamine B, respectively.

### Statistical Analysis

All the statistical analyses were performed by using SAS version 9.4 (SAS institute, Cary, North Carolina, United States). Mean and standard deviation or median and IQR (interquartile range) were computed for maximum sealer, MFI, and SFI according to normality of the variable. Analysis of variance (ANOVA) or Kruskal–Wallis H test were used to compare average of the variables among AHC, AHS, and EMS group according to normality of the variable. For multiple comparison correction, Bonferroni adjustment was used. The level of significance was set at *p* ≤0.05. A post hoc power analysis was performed to confirm the appropriateness of the sample size for mean comparison among AHC, AHS and MFI groups using significance level of α = 0.05 and power = 0.8.

## Results

**Fig. 1:**
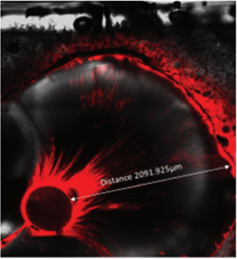
Measurements of the maximum sealer penetration depth.

**Fig. 2:**
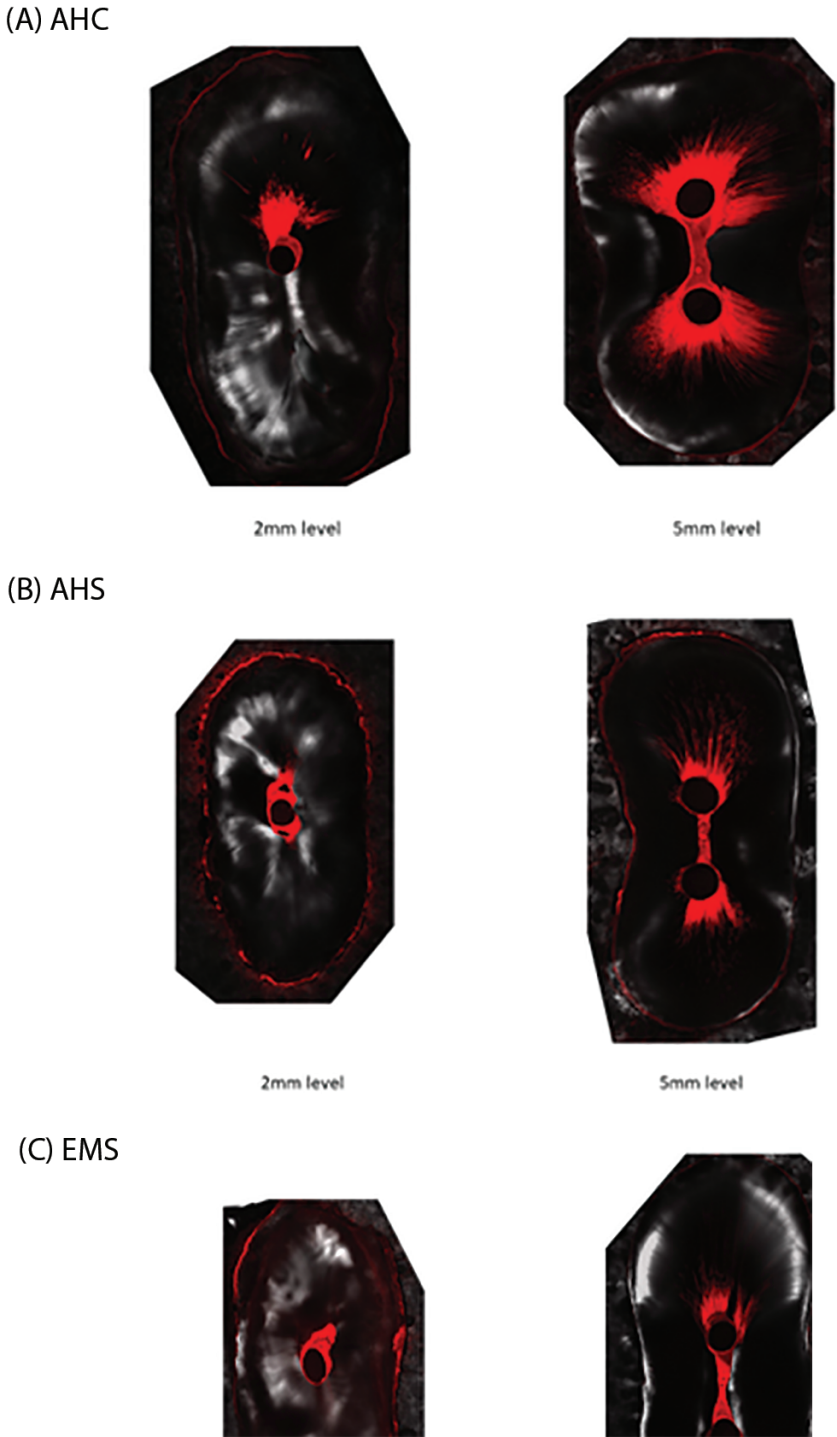
(A-C) Representative confocal laser scanning microscopy images at the 2-mm level and 5-mm level from the apex (×5)

**Fig. 3:**
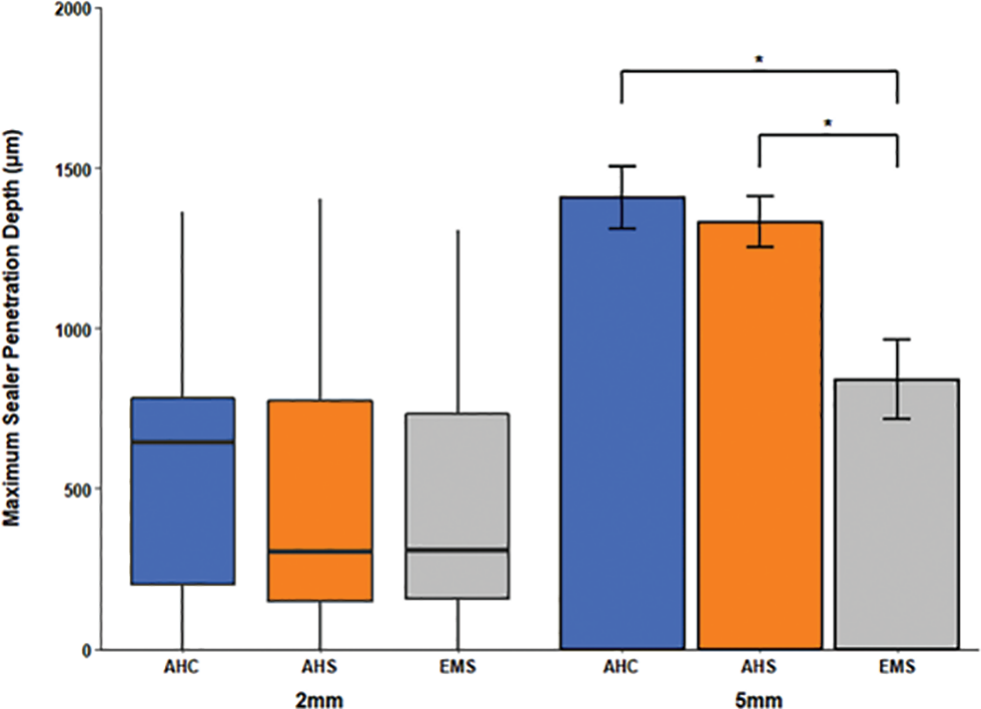
Comparison of maximum sealer penetration depth values among experimental groups (unit = μm). For 2-mm level, median and interquartile ranges are shown. For 5-mm level, mean ± standard errors are shown. At the 2-mm level, there was no statistically significant difference among the groups (Kruskal–Wallis H test). At the 5-mm level, the Endoseal MTA + single cone technique with ultrasonic activation group showed statistically significantly lower values than the AH Plus + continuous wave and AH Plus + single cone technique groups ( *p* < 0.01, adjusted using Bonferroni method after analysis of variance, denoted as *). Sample numbers in each group were 15.

The maximum sealer penetration depth into the dentinal tubules, as well as MFI and SFI, were measured by using the ZEN software program ( Fig. 1 ). Fig. 2 shows representative CLSM images at the 2-mm level and 5-mm level from the apex, where the AHS group had more voids in the isthmus area than the other groups. In all groups, the sealer penetration depth was higher at the 5-mm level than the 2-mm level. At the 5-mm level, the EMS group showed the lowest value compared with AHC and AHS group ( *p* < 0.01, adjusted using Bonferroni method after ANOVA; Fig. 3 ). At the 2-mm level, there was no statistically significant difference among all three groups (Kruskal–Wallis H test).

**Fig. 4:**
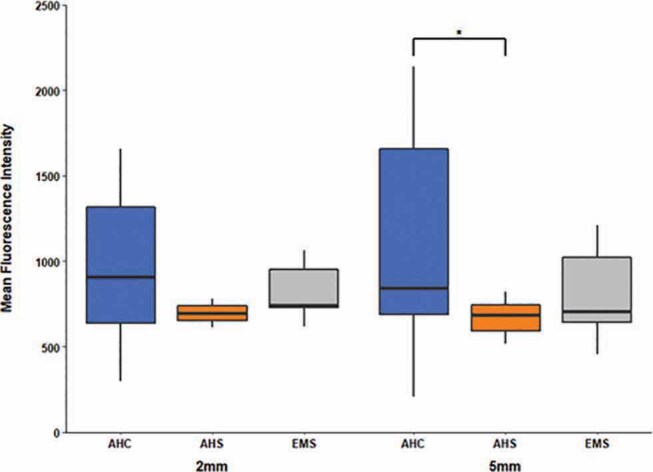
Comparison of unitless mean fluorescence intensity values among experimental groups. For all levels, median and interquartile ranges are shown. At the 2-mm level, there was no statistically significant difference among groups (Kruskal–Wallis H test). At the 5-mm level, the AHC group showed a statistically significantly higher value than the AHS group ( *p* = 0.027, adjusted using Bonferroni method after Kruskal–Wallis H test, denoted as *). Sample numbers in each group were 15.

The MFI values, which reflect the mean intensity of rhodamine B in the specimen, at the 5-mm level were higher than those at the 2-mm level in all groups. At the 5-mm level, the AHC group showed higher values than the AHS group ( *p* = 0.027, adjusted using Bonferroni method after Kruskal–Wallis H test; Fig. 4 ). At the 2-mm level, there was no statistically significant difference among all three groups (Kruskal–Wallis H test).

**Fig. 5:**
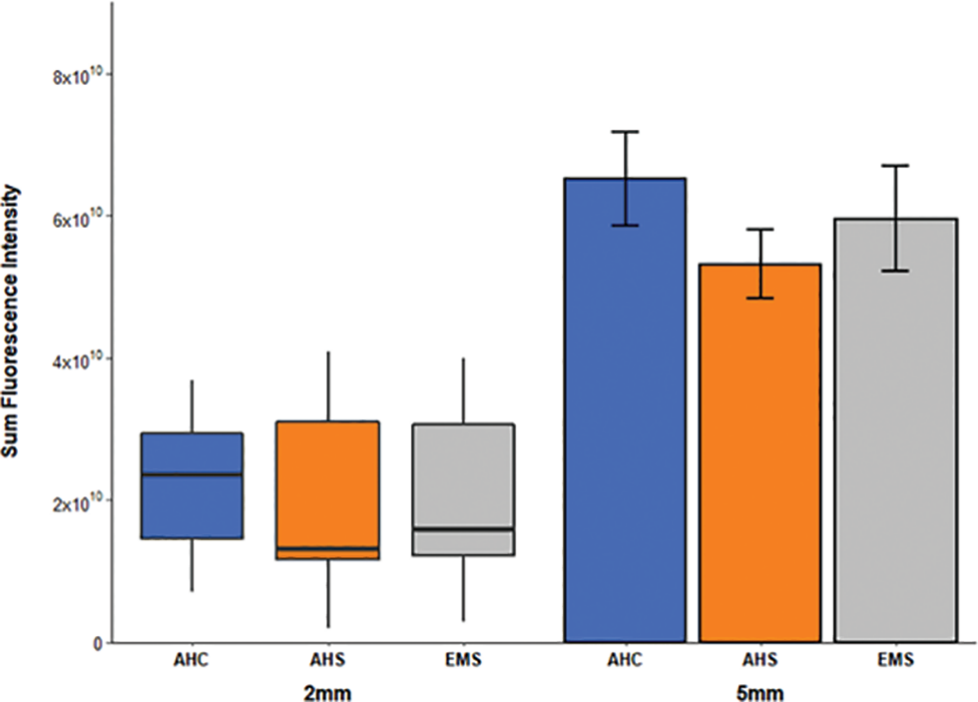
Comparison of unitless sum fluorescence intensity values among experimental groups. For 2-mm level, median and interquartile ranges are shown. For 5-mm level, mean ± standard errors are shown. There was no significant difference among groups at all levels (Kruskal–Wallis H test for 2-mm level and analysis of variance for 5-mm level). Sample numbers in each group were 15.

The SFI values, which denote the total intensity of rhodamine B in the specimen, at the 5-mm level were higher than those at the 2-mm level in all groups. The AHC group showed the highest SFI value at all levels and the AHS group showed the lowest value at all levels, but there was no statistically significant difference among all three groups at either level ( Fig. 5 , Kruskal–Wallis H test for the 2-mm level and ANOVA for the 5-mm level). Table 1 shows the distribution of variables at 2-mm and 5-mm levels in each group.

**Table 1:** Distribution of variables at 2-mm and 5-mm levels in each group

Depth	Group	MFI	SFI	Penetration Depth
2 mm	AHC	906 (636–1,317)	23,467,605,104 (14,485,802,363–29,316,498,831)	644.8 (203.3–781.9)
	AHS	695 (654–739)	13,037,655,355 (11,581,334,973–31,027,198,735)	303.2 (146.8–775.6)
	EMS	740 (728–951)	15,765,907,185 (12,148,750,687–30,631,958,607)	307.6 (158.6–731.5)
5 mm	AHC	840 (690–1,656)	65,051,196,506 ± 6,566,107,330	1,405.6 ± 96.5
	AHS	681 (592–745)	53,162,244,503 ± 49,16,702,335	1,330.7 ± 79.7
	EMS	704 (643–1,021)	59,548,225,221 ± 7,347,856,908	840 ± 122.8
Abbreviations: AHPC, AH Plus + continuous wave; AHPS, AH Plus + single cone technique; EMS, Endoseal MTA + single cone technique with ultrasonic activation group; MFI, mean fluorescence intensity; SFI, sum fluorescence intensity. Note: Variables are presented as mean ± standard error or median (interquartile ranges) according to normality of the variable.				

## Discussion

In the current study, sealer penetration was evaluated under CLSM. In previous research, scanning electron microscopy (SEM), 19 20 light microscopy (LM), 17 and CLSM have been used to evaluate the sealing ability of root canal sealers. 21 22 23 In contrast to SEM, CLSM produces fewer artifacts and does not promote specimen dehydration. 24 It also provides a detailed view of interfacial adaptation and the distribution of sealers using fluorescence. 19 With LM, it is impossible to differentiate the sealer from the dentin. 25 CLSM has high contrast, which allows a proper analysis of the sealer in the dentinal tubules. 26 27 The sealer should be labeled with rhodamine B because it allows for the identification of sealers within the dentinal tubules 28 and has no effect on its physical properties, as long as a small amount of dye (less than 0.2%) is used. 5 29 Rhodamine B is known to show a powerful affinity for moisture and has less affinity for calcium in the sealer composition. As a result, it can penetrate more deeply into the dentinal tubules, giving inaccurate results. 30 However, Patel et al 5 tested the degree of tubule penetration of the sealer according to the presence or absence of rhodamine B dye. They said that the penetration results were similar regardless of the rhodamine dye in the experiment, and that the presence of the dye could lead to false results. The possibility of false results due to leaching of rhodamine B from the sealers was therefore excluded.

The present study showed that the maximum sealer penetration depth, MFI, and SFI were higher at the 5-mm level than at the 2-mm level in the experimental groups. This result is in accordance with other studies that found deeper dentinal tubule penetration of resin-based sealer into the dentinal tubules at the 5-mm level than at the 3-mm and 1-mm levels. 21 31 This finding is also similar to the results reported by McMichael et al, 32 who found that the BC Sealer penetration depth was significantly higher at the 5-mm level than at the 1-mm level. This can be explained by the fact that the number and diameter of dentinal tubules decrease toward the apical end of the root canal. 23 Furthermore, there is more sclerotic dentin and anatomical variation in the apical third than in the coronal third. 33 Another possible reason is that it is difficult to extend the endodontic instrument to the apical third, so it is challenging to apply sufficient irrigation, 33 which may lead to less removal of the smear layer when compared with the coronal third of the canal. Also, it is difficult to access the apical third using filling devices such as heat carriers, so that the filling efficiency might be lower than in the coronal third area.

In this study, although the highest measurements were obtained in the AHC group, there were no statistically significant differences among all groups at the 2-mm level ( *p* > 0.05). Furthermore, the AHS group, without application of heat and pressure, showed the lowest MFI and SFI values. It is well known that the continuous wave technique with warm GP produces consistently dense, dimensionally stable, three-dimensional root canal fillings. Therefore, it enables canal irregularities to be filled more effectively than is possible with cold GP. 34 Furthermore, this technique provides better sealing against coronal microbial penetration than the single cone filling technique. 35 Commonly, the AH Plus sealer is suitable for the continuous wave technique because it is heat-resistant. It has been reported that AH Plus sealer showed acceptable changes in physical properties (setting time and flow) at a high temperature. 36 On the contrary, significant reductions in the setting time and flow in the silicate-based sealer were found. 9

However, the continuous wave technique could generate a certain amount of pressure on the radicular dentin, causing vertical root cracks or fractures. 37 In this technique, heat is transferred only approximately 2 to 3 mm from the area, where the heat device is applied and the GP may be softened. 38 The apical third area is important for the success of the root canal treatment. 39 In this study, at the 2-mm level, there was no significant difference among the three groups. These results show that sealing efficacy of the continuous wave technique can be lowered in the apical area where heat and pressure are difficult to apply, especially in curved canals and irregularly shaped canals (e.g., C-shaped or type II canals).

In particular, C-shaped canals, in which two or more canals are connected with mesh or fin-shaped structures in the form a “C” letter, are prevalent in Asian populations. 40 These canals usually have very thin lingual walls; therefore, there is a higher risk of root perforation or root fracture during endodontic treatment. Gok et al 41 found more void areas when the continuous wave technique was used than when the cold lateral filling technique was used in the C1 root canal configuration (Fan’s classification) at 2 mm. It is also difficult to fill in the apical part of the canal curvature. Likewise, type II (ribbon-shaped) canals have thin isthmus areas. Therefore, the continuous wave technique seems to be more limited in type II canal and complex root canal structure such as isthmus.

Endoseal MTA, which is a recently launched calcium silicate-based sealer, uses the single cone technique with ultrasonic activation. It is thought that the single cone technique takes less time and is less likely to cause cracks or damage surrounding tissues because there is no need for heat and vertical pressure. 42 For effective dentinal wall adaptation, the manufacturer recommends applying ultrasonic power directly to the GP cone when using Endoseal. Some study reported that Endoseal MTA using ultrasonic vibration showed better sealer distribution than the AH Plus sealer with the continuous wave technique. 43 With ultrasonic activation, the Endoseal MTA obturates complex anatomical variations more completely and has better filling quality, with fewer voids, than when ultrasonic activation is not used. 42

Although the EMS group showed the lowest maximum sealer penetration depth value at the 5-mm level in this study. In many other studies, 42 44 45 a calcium silicate-based sealer showed good flowability and sufficient filling quality regardless of the filling technique. In contrast, this study did not show higher penetration depth values. Endoseal MTA is a premixed injectable sealer. In this experiment, however, the GP tip was coated with the sealer that was mixed in the pad, not syringe injection, since Endoseal MTA should be labeled with rhodamine B. This may be an explanation for why the EMS group showed the lowest maximum sealer penetration depth. In addition, ultrasonic activation with P-5 Newtron XS was also completed in this study, but it was set on “3” in the green mode, which is thought to be insufficient for sealer distribution. In some recent papers, Endoseal was reported that it showed the best filling ability without voids among the sealers used when the Endoseal was injected into the canal with a syringe and activated ultrasonically set on power “8.” 46

However, the EMS group showed markedly lower maximum sealer penetration depth values, whereas this was not the case for MFI and SFI values. The maximum sealer penetration depth value refers directly to the maximum degree to which the sealer can penetrate. Therefore, even if the overall sealer penetration does not show higher values, the presence of one or two areas with deep penetration may yield large values. Because the size of the specimen is different for each tooth size and shape, high maximum penetration depth values may be found for large or elongated teeth, which may lead to errors, especially when the sample size is small. It is thought that the EMS group showed the lowest maximum penetration depth value for this reason. Therefore, MFI and SFI values, which show the overall sealer penetration, are more meaningful results than the maximum sealer penetration depth value.

In this experiment, although not recommended by the manufacturer, the AH Plus sealer was also applied by using the single cone technique, and the AHC group showed a higher sealer penetration value than AHS group. The AHS group showed higher maximum penetration depth at the 5-mm level than the EMS group and similar MFI and SFI values to the EMS group. However, the AHS group had more voids in the isthmus area, underscoring the limits of using this technique without heat or vertical pressure. Therefore, for epoxy resin-based sealers, the continuous wave technique is recommended as a better filling technique in terms of filling efficacy than the single cone technique. In fact, a study on the physical properties of AH Plus reported that applying heat reduced the setting time and increased the film thickness. The flow did not change even when heat was applied. By applying pressure to the softened GP by applying heat, the adaptability of AH Plus and GP to the dentin wall increases. In other words, in the continuous wave technique, heat and pressure work together to obtain an effect. Further experiment using various sealers and comparing various methods of delivering, the sealer into the canal is likely to be necessary. Since the sealer penetration depth evaluated in this study is only one factor of the sealability of the sealer, it does not mean the whole and it is thought that additional evaluation of various physical properties is also necessary. In addition, some studies reported that there was no significant correlation between sealability and sealer penetration into dentinal tubules. 47 48 Other factors related to sealability need to be evaluated. There were no differences in penetration depth into dentinal tubule especially at 2-mm level among groups tested, and the null hypothesis was accepted.

## Conclusion

The AH Plus group with the continuous wave technique (AHC group) showed the highest dentinal tubule penetration, but in the apical area, which is the most important part for the success of root canal therapy, there was no statistically significant difference from other sealer groups. Therefore, in areas such as the apical 2 mm of premolars with type II canals, which are difficult to access using instruments such as heat carriers various approaches may be required accordingly.
